# Comparative Effects of High-Molecular-Weight Chitosan and Chitosan Oligosaccharide Lactate on Metabolic Adjustment and Salinity Tolerance in *Cucumis sativus* L.

**DOI:** 10.3390/molecules31183306

**Published:** 2026-09-17

**Authors:** Barbara Hawrylak-Nowak, Maria Stasińska-Jakubas, Jan Sawicki, Renata Matraszek-Gawron, Weronika Jarmulska, Magdalena Czelej-Wrzesień, Sławomir Dresler

**Affiliations:** 1 Department of Botany and Plant Physiology, University of Life Sciences in Lublin, Akademicka 15, 20-950 Lublin, Poland; barbara.nowak@up.edu.pl (B.H.-N.); renata.matraszek@up.edu.pl (R.M.-G.); weronika.jarmulska@up.edu.pl (W.J.); 2 Department of Analytical Chemistry, Medical University of Lublin, Chodźki 4a, 20-093 Lublin, Poland; jan.sawicki@umlub.edu.pl (J.S.); slawomir.dresler@umlub.edu.pl (S.D.); 3 Department of Vascular Surgery and Angiology, Medical University of Lublin, Staszica 16, 20-081 Lublin, Poland; magdalena.czelej@umlub.edu.pl; 4 Department of Plant Physiology and Biophysics, Maria Curie-Skłodowska University, Akademicka 19, 20-033 Lublin, Poland

**Keywords:** biostimulants, salt stress, chemical form–biological activity relationship, metabolic responses, tricarboxylic acid (TCA) cycle, ion homeostasis

## Abstract

Salinity is one of the most harmful abiotic factors limiting crop productivity. Chitosan is a natural polysaccharide biostimulant able to alleviate salt stress, but its efficiency depends on its physicochemical properties, which remain insufficiently understood. The present study aimed to determine whether two chemically distinct forms of chitosan, i.e., high-molecular-weight chitosan (ChT) and chitosan oligosaccharide lactate (ChL), differentially mitigate the adverse effects of NaCl-induced salinity in cucumber (*Cucumis sativus* L.). Plants were grown hydroponically under control conditions or in the presence of 50 mmol L^−1^ NaCl, with or without medium-applied ChT or ChL (10 mg L^−1^). Salinity reduced growth and the accumulation of photosynthetic pigments, whereas chlorophyll fluorescence parameters remained largely unaffected, indicating limited impairment of PSII photochemistry. Chitosan, particularly the ChT form, partially alleviated NaCl-induced growth inhibition; ChT increased shoot and root fresh weight and leaf area under salt stress, whereas the effects of ChL were not statistically significant. Chitosan also enhanced free proline accumulation and was associated with changes in selected tricarboxylic acid (TCA) cycle intermediates, particularly malate and α-ketoglutarate, indicating an association with altered central carbon metabolism under salt stress. In contrast, neither chitosan form substantially improved the ionic status of cucumber, as excessive Na accumulation and reductions in K concentration and the K/Na ratio persisted despite treatment. These findings suggest that the beneficial effects of chitosan, particularly ChT, in salt-stressed cucumber were associated more closely with metabolic and osmotic responses than with restoration of ion homeostasis or direct protection of the photosynthetic apparatus, with the two chitosan forms showing different response patterns under the experimental conditions used.

## 1. Introduction

Soil salinity remains one of the most limiting abiotic stresses for crop production, particularly in regions where intensive irrigation coincides with low precipitation levels. Excessive accumulation of soluble salts in the rhizosphere disrupts plant–water relations and causes ion imbalances, which ultimately leads to osmotic stress. The oxidative stress resulting from these disruptions contributes to reduced growth and yield [1,2,3,4]. A key feature of salinity stress is excessive Na accumulation combined with reduced K uptake, leading to a decreased K/Na ratio, which is widely considered one of the major causes of salinity toxic effects [5,6]. Considering the rapid expansion of salinized agricultural soils [7], the development of environmentally friendly strategies capable of preserving metabolic homeostasis under saline conditions has become an important objective of sustainable crop production. Primary carbon metabolism, particularly the tricarboxylic acid (TCA) cycle, is crucial for plant adaptation to salinity, since it links carbon metabolism, energy production and osmotic adjustment [8,9]. Therefore, changes in the pools of TCA-cycle intermediates provide valuable insight into metabolic reprogramming underlying changes in plant stress tolerance.

Chitosan, a deacetylated derivative of chitin, is one of the natural polymer biostimulants that has attracted growing attention in this context. It is appreciated for its favorable properties, such as antioxidant and antimicrobial activity, non-toxicity, biodegradability, and biocompatibility [10,11]. Although chitosan was originally studied mainly in relation to plant–pathogen interactions, its role has since been extended to activation of plant defense responses and regulation of metabolic plasticity, which enhance tolerance to abiotic stresses, including salinity, drought and suboptimal temperatures. Some studies showed that chitosan can enhance the efficacy of antioxidant systems, improve osmotic adjustment through the accumulation of osmolytes, and stabilize photosynthetic performance under stress conditions [12,13,14].

The biological activity of chitosan is significantly influenced by its chemical characteristics, which include the degree of deacetylation (DD), molecular weight (MW), and formulation type (e.g., chitosan hydrochloride, chitosan acetate, nanoparticles), which impact its solubility and charge density [15,16]. These features determine the form–activity relationship of chitosan, influencing its interactions with plant cell membranes, perception by membrane receptors, action on nuclear chromatin components, and the subsequent activation of signaling pathways that lead to metabolic reprogramming [17,18]. Nevertheless, most studies on chitosan-mediated salinity tolerance have relied on a single, often insufficiently characterized chitosan reagent, without reporting key physicochemical parameters such as molecular weight or degree of deacetylation [19,20], making it difficult to determine how specific physicochemical properties affect its bioactivity [21].

Cucumber (*Cucumis sativus* L.) is one of the most commercially important vegetables worldwide and is also known to be sensitive to soil salinity, which disrupts its physiological processes and reduces yield [22,23]. The majority of the available results regarding the biological activity of chitosan in cucumber are restricted to foliar applications of this polymer in plants cultivated in soil [24] or were focused on other types of abiotic stresses [25]. Although there is little information regarding root-applied chitosan in plants exposed to NaCl, recent studies indicate that root-zone or nutrient-solution application may also effectively modulate plant responses to salinity [26]. Also, although increases in growth and osmolyte accumulation, and in some cases ion homeostasis, have been reported, the impact of chitosan on central carbon metabolism, particularly with regard to organic acids associated with the tricarboxylic acid (TCA) cycle, has received insufficient attention. There is a lack of integrative studies that combine growth analysis, photosynthetic parameters, osmolyte accumulation, organic acid profiles and changes in mineral status within a single experimental system comparing different chitosan formulations.

The physicochemical characteristics of chitosan, including molecular weight, degree of deacetylation, chemical form and solubility, may influence its biological activity under stress. Therefore, we hypothesized that the two chemically distinct chitosan forms, i.e. high-molecular-weight chitosan (ChT) and chitosan oligosaccharide lactate (ChL), differ in their ability to mitigate the negative consequences of excessive salinity in cucumber. Taking into account these knowledge gaps, this study provides new insights into how chemically distinct chitosan forms may differentially influence plant responses to abiotic stress. This knowledge can help optimize chitosan-based biostimulants and has the potential to contribute to the establishment of sustainable, salt-tolerant agriculture.

## 2. Results

### 2.1. Mineral Nutrient Status and Ionic Homeostasis

The experiment involved hydroponic cucumber cultures supplemented with two chemically distinct chitosan forms under control and NaCl-induced salinity conditions. Salinity profoundly disrupted mineral nutrition, resulting in a substantial increase in Na accumulation and a marked decrease in K content in roots and shoots. Consequently, the K/Na ratio was severely reduced under salinity from above 100 in the control and chitosan-treated plants to nearly 1 (in roots) or less than 1 (in shoots) of NaCl-exposed plants (Table 1 and Table 2).

The Mg and Ca levels in roots were significantly reduced under salinity, while P, S, Cu, and Mn contents increased in plants subjected to NaCl (Table 1). Also, Mg, Ca, P, and Mo contents were significantly reduced in shoots (Table 2). Plants treated with chitosan alone (ChT or ChL) generally maintained a mineral profile similar to the control, with high K levels and low Na content. Thus, the K/Na ratio remained high in shoots and roots and did not differ significantly from control plants (Table 1 and Table 2).

Under salinity, the application of both chitosan forms only slightly modified ion composition; however, they did not restore K levels or reduce Na accumulation. As a consequence, the K/Na ratio remained low in NaCl-treated plants, regardless of chitosan presence. The slight improvements in selected elements under NaCl + chitosan treatments were found in shoots for Ca and in roots for Mo; although these effects were not consistent across all treatments (Table 1 and Table 2). Therefore, the effect of chitosan application on the mineral composition of cucumber under salinity was limited and did not result in a substantial restoration of the measured mineral elements.

### 2.2. Photosynthetic Performance and Pigment Composition

**Figure 1:**
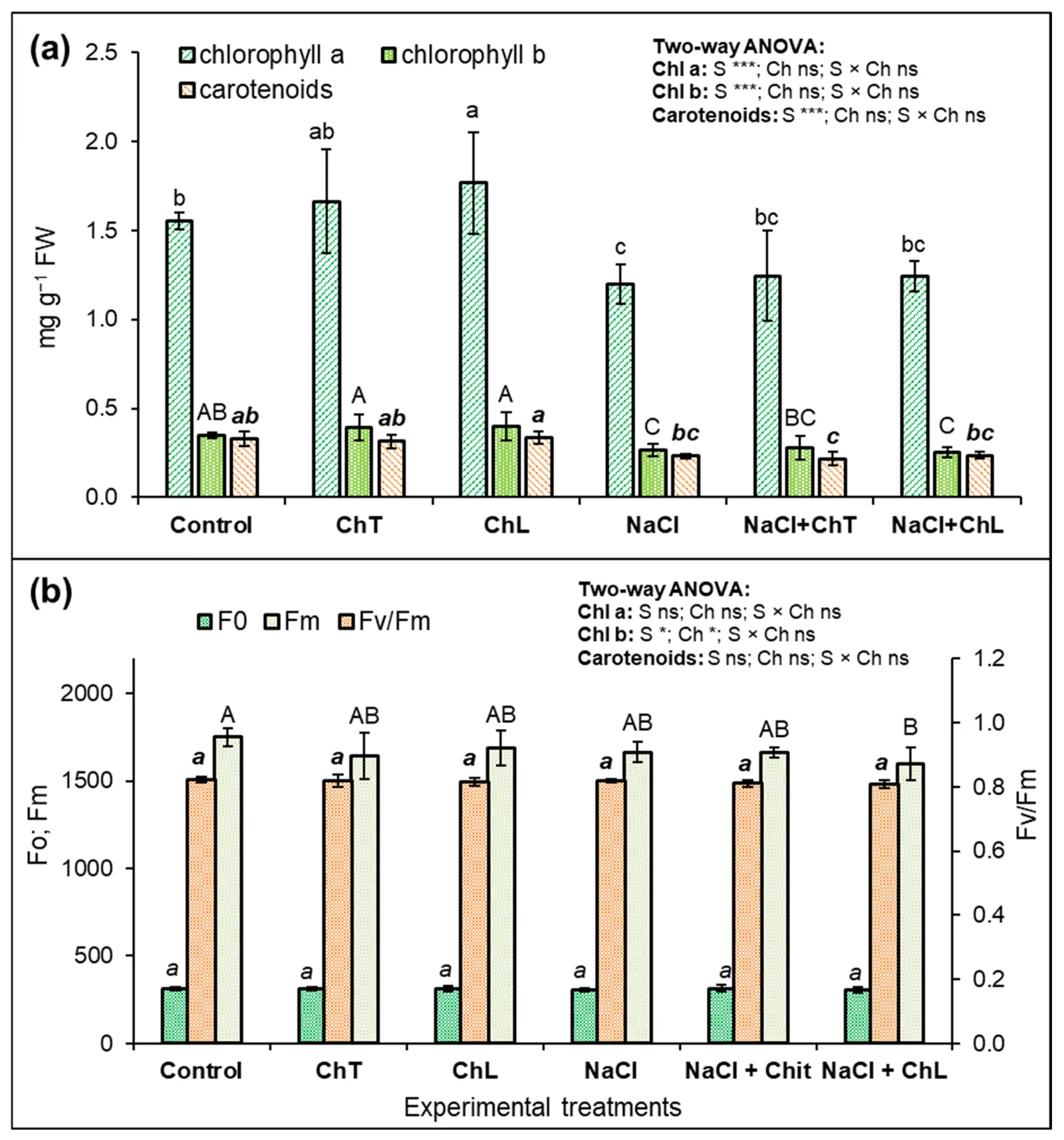
Effect of ChT or ChL application on the photosynthetic pigments content (**a**) and selected parameters of chlorophyll a fluorescence (**b**) of *C. sativus* grown under NaCl-induced stress. Means (±SD, *n* = 3 for photosynthetic pigments, *n* = 9 for fluorescence parameters) followed by different letters above the bars indicate statistically significant differences between treatments for the respective parameter (*p* < 0.05, Tukey’s test). Lowercase, italic, and capital letters are used to distinguish the statistical comparisons performed for the individual parameters and do not represent different levels of significance. Statistical comparisons should be made only among groups annotated within the same parameter; groups sharing the same letter do not differ significantly. Two-way ANOVA: salinity (S), chitosan treatment (Ch), S × Ch interaction; ns—not significant, * *p* < 0.05, *** *p* < 0.001.

Exposure to NaCl resulted in a significant decrease in photosynthetic pigment content, including chlorophyll a, chlorophyll b and carotenoids (Figure 1a). In non-stressed plants, application of ChL promoted chlorophyll a accumulation, but under salinity its presence did not improve photosynthetic pigment levels compared to salt-stressed plants grown without chitosan. Although plants treated with ChT or ChL exhibited slightly higher mean pigment levels compared with NaCl alone treatment, these differences were not statistically significant (Figure 1a). Even when only the treatments containing NaCl were considered, the differences were not significant.

In turn, the chlorophyll a fluorescence parameters exhibited a generally high degree of stability across the experimental treatments (Figure 1b). No significant main effects or interactions were detected for the minimum fluorescence (Fo) or the maximum quantum efficiency of PSII photochemistry (Fv/Fm). In contrast, the maximum fluorescence (Fm) was significantly affected by both salinity and chitosan treatment, whereas the salinity × chitosan interaction was not significant. Tukey’s post hoc test showed that control plants were characterized by significantly higher Fm values than those subjected to 50 mM NaCl in combination with ChL, while no other pairwise comparisons were significant (Figure 1b). Thus, although Fm showed treatment-related variation, the unchanged Fo and Fv/Fm values indicate that these changes were not accompanied by a significant alteration in the maximum quantum efficiency of PSII under the applied experimental conditions. 

Because salinity-induced impairment of photosynthesis is frequently associated with metabolic adjustments, the accumulation of free proline and organic acids related to the TCA cycle was further investigated.

### 2.3. Metabolic Adjustment: Free Proline and Organic Acid Accumulation

**Figure 2:**
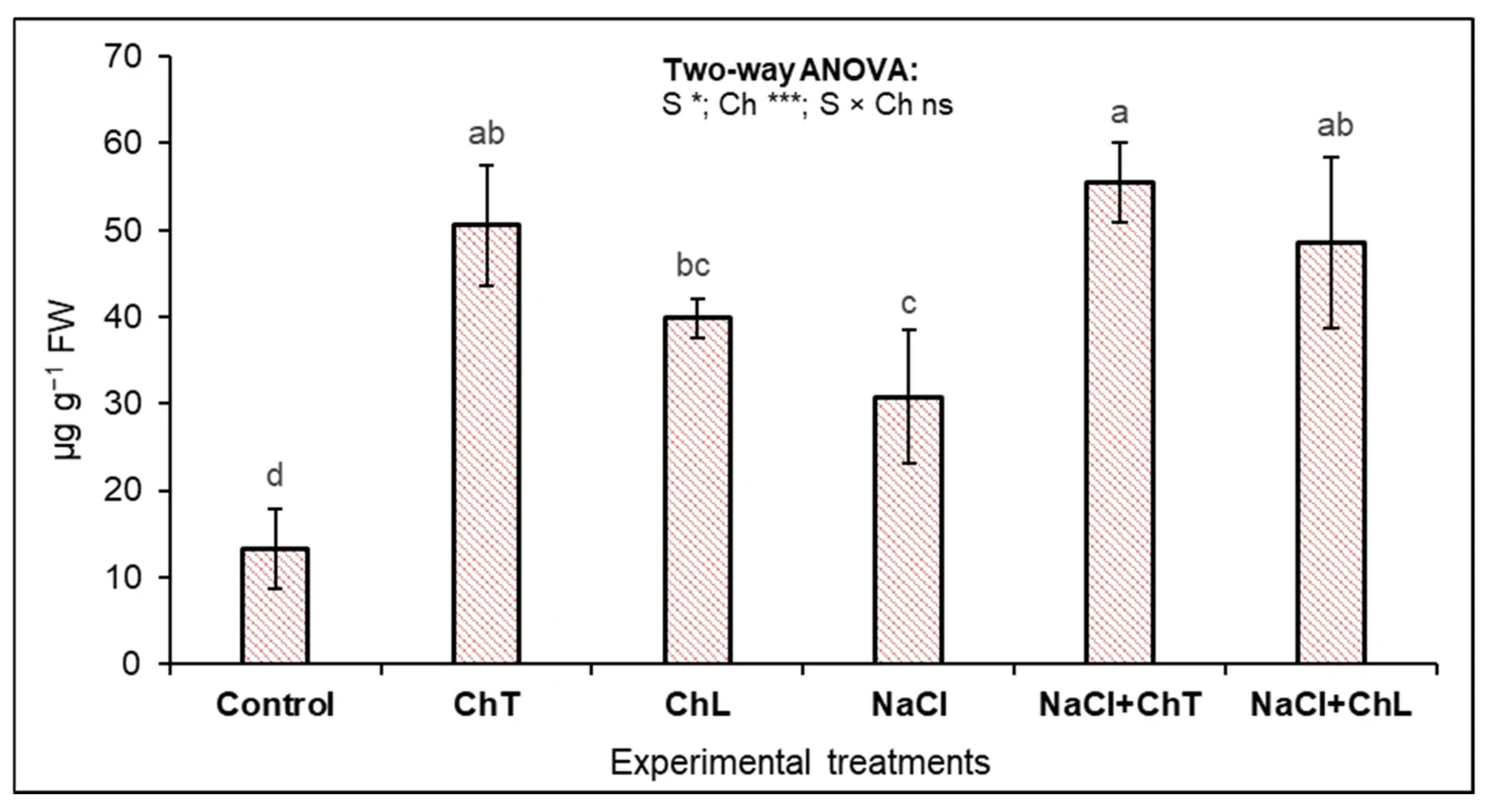
Effect of chitosan application on free proline accumulation in the leaves of *C. sativus* grown under NaCl-induced stress. Means (±SD, *n* = 4) with different letters above the bars are significantly different (*p* < 0.05, Tukey’s test). Two-way ANOVA: salinity (S), chitosan treatment (Ch), S × Ch interaction; ns—not significant, * *p* < 0.05, *** *p* < 0.001.

The concentration of free proline in leaves increased by more than twice under salt stress conditions (Figure 2), which reflects its role as a compatible osmolyte in the plant salt stress response. Application of chitosan further elevated proline levels under salinity, suggesting that enhanced accumulation of this amino acid may contribute to osmotic adjustment under salinity. Interestingly, in non-stressed plants, application of chitosan, especially in the form of ChT, also markedly elevated proline levels even more than salinity itself. The two chitosan forms showed somewhat different patterns of proline accumulation, although these differences should not be interpreted as evidence of a statistically significant direct difference between ChT and ChL.

**Figure 3:**
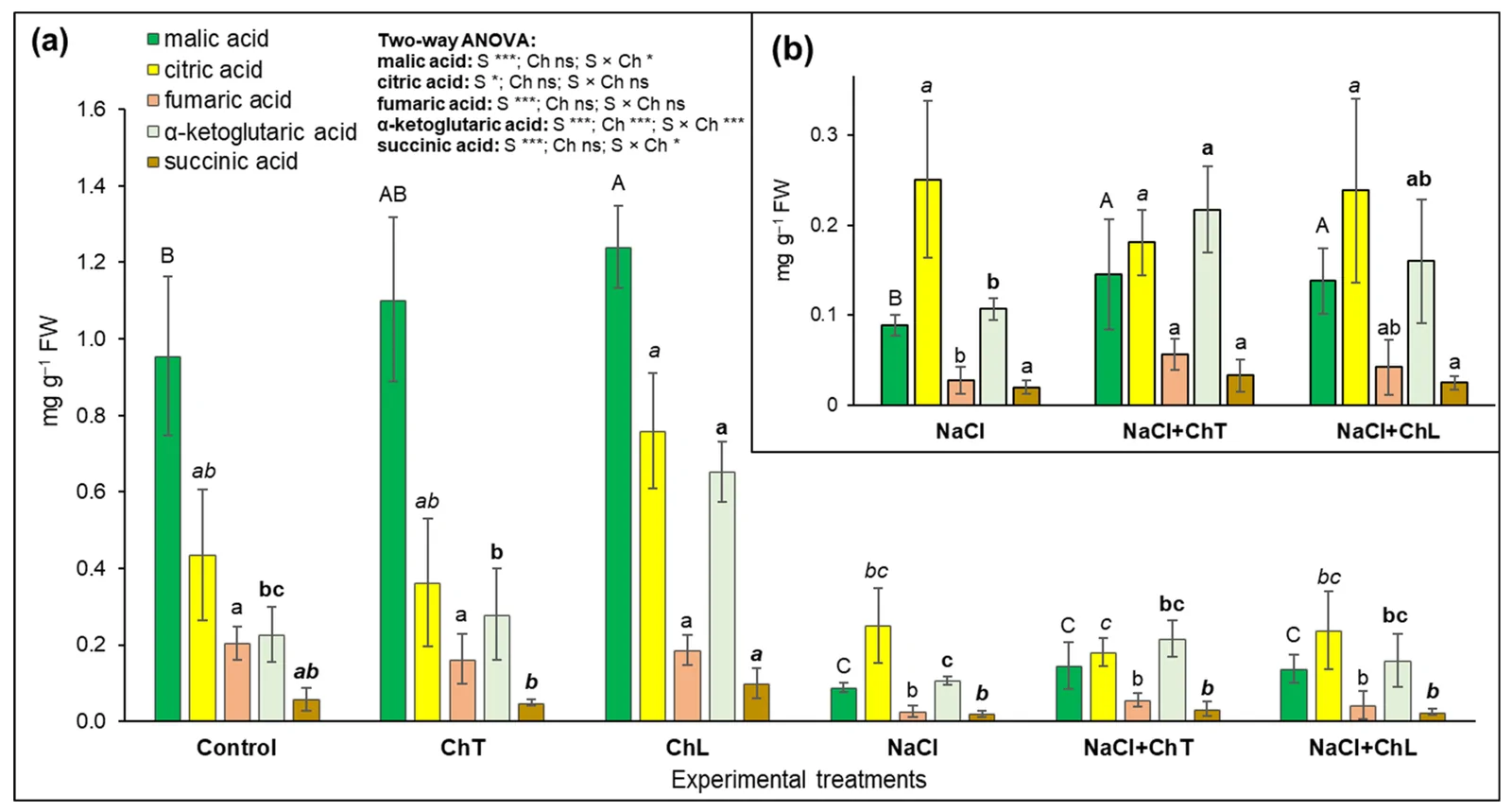
Effect of ChT or ChL application on the content of organic acids related to the TCA cycle in *C*. *sativus* grown under NaCl-induced stress. Means (±SD, *n* = 4) with different letters (lowercase, italic, or capital) above the bars indicate statistically significant differences between treatments for concentrations of individual organic acids (*p* < 0.05, Tukey’s test) (**a**). Panel (**b**) presents the results of the complementary one-way ANOVA performed exclusively for the NaCl-containing treatments. Lowercase, italic, and capital letters are used to distinguish the statistical comparisons performed for the individual parameters and do not represent different levels of significance. Statistical comparisons should be made only among groups annotated within the same parameter; groups sharing the same letter do not differ significantly. Two-way ANOVA: salinity (S), chitosan treatment (Ch), S × Ch interaction; ns—not significant, * *p* < 0.05, *** *p* < 0.001.

The concentrations of organic acids related to the TCA cycle in the leaves of cucumber were significantly affected by salinity (Figure 3a). Two-way ANOVA showed a significant main effect of salinity on all analysed organic acids. Chitosan treatment affected α-ketoglutarate concentrations, while a significant salinity × chitosan interaction was detected for α-ketoglutarate, malate, and succinate. Salt stress caused a substantial decline in the concentration of all organic acids compared with both control and chitosan-treated plants. Among the TCAs, malate and α-ketoglutarate exhibited the most pronounced treatment-dependent responses, while changes in citrate, fumarate and succinate were relatively slighter. Application of both forms of chitosan under non-saline conditions maintained or even increased malate levels relative to the control (Figure 3a). 

Because the dominant effect of salinity masked differences between the two chitosan formulations, an additional analysis restricted to NaCl treatments was performed (Figure 3b), allowing the treatment-specific responses to be resolved. Under salinity, both ChT and ChL partially alleviated the NaCl-induced reduction in malate concentrations. Although neither chitosan form completely restored malate concentration to the control level, both clearly mitigated the inhibitory effect of salinity. An even more pronounced response was observed for α-ketoglutarate. In NaCl-treated plants, only ChT caused an increase in α-ketoglutarate and fumarate concentration relative to NaCl alone, indicating partial maintenance of selected TCA-cycle metabolite pools under saline conditions (Figure 3b). In contrast, the concentrations of citrate and succinate showed less pronounced fluctuations. Although some changes were observed after chitosan application, particularly under non-saline conditions, these responses were generally smaller than those recorded for malate or α-ketoglutarate (Figure 3a,b).

Overall, the two chitosan preparations showed partially different patterns of TCA-cycle intermediates responses, particularly under saline conditions. Notably, the beneficial effects of chitosan on organic acids related to the TCA cycle became evident only after analysing the saline treatments separately, highlighting the importance of evaluating treatment effects within a uniform stress background.

### 2.4. Plant Growth Response to Chitosan in Non-Stress and Salt Stress Conditions

**Figure 4:**
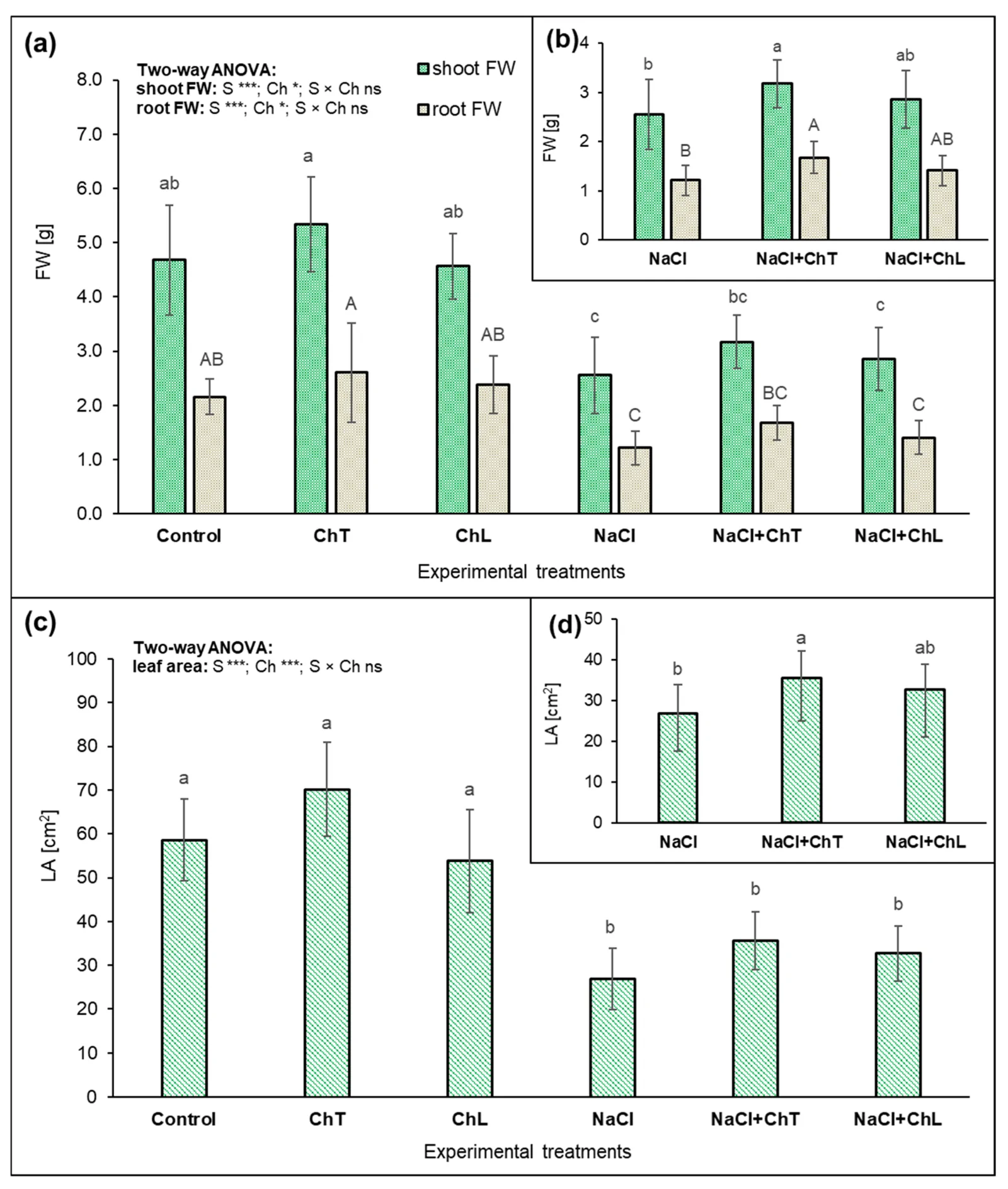
Changes in the fresh weight of shoots and roots (**a**,**b**) and the leaf area (**c**,**d**) of *C. sativus* after the application of ChT or ChL under NaCl-induced stress. Means (±SD, *n* = 9) with different letters above the bars (lowercase or capital) indicate statistically significant differences between treatments for individual plant organs (*p* < 0.05, Tukey’s test). Panels (**b**) and (**d**) present the results of the complementary one-way ANOVA performed exclusively for the NaCl-containing treatments. Lowercase, italic, and capital letters are used to distinguish the statistical comparisons performed for the individual parameters and do not represent different levels of significance. Statistical comparisons should be made only among groups annotated within the same parameter; groups sharing the same letter do not differ significantly. Two-way ANOVA: salinity (S), chitosan treatment (Ch), S × Ch interaction; ns—not significant, * *p* < 0.05, *** *p* < 0.001.

Salinity strongly inhibited the growth of cucumber, causing a decrease in shoot and root fresh weight (FW) as well as leaf area (Figure 4a,c). Two-way ANOVA confirmed a highly significant main effect of salinity on shoot FW, root FW and leaf area. Chitosan treatment also had a significant main effect on these parameters, whereas the salinity × chitosan interaction was not significant. Plants exposed to NaCl showed a strong reduction in biomass compared with the control, with mean shoot FW decreasing from 4.68 g in the control plants to 2.55 g under salt stress (45% reduction). A similar trend was observed for root FW, which declined to a similar extent. The most sensitive parameter to salinity was the leaf area (LA), which decreased by more than half. Application of chitosan alone did not negatively affect growth and, in some cases, slightly stimulated biomass accumulation. Plants treated with ChT showed a slightly higher shoot FW and LA compared with the control, while ChL maintained FW values close to control (Figure 4a,c).

Under salinity, chitosan partially alleviated the inhibitory effects of NaCl. In the complementary one-way analysis restricted to NaCl-containing treatments (Figure 4b,d), the NaCl + ChT treatment resulted in significantly higher shoot and root FW and LA compared with the NaCl-alone treatment. Under these conditions, the shoot and root FW increased by 24% and 38% (Figure 4b), respectively, and LA increased by 32% (Figure 4d) compared with the NaCl-alone treatment. A weaker mitigation effect was observed for NaCl + ChL, but the differences were not statistically significant. These results indicate that chitosan, particularly in the ChT form, alleviated the negative effects of salinity on shoot and root growth and leaf expansion to some extent. The two preparations showed different response patterns, with a significant improvement relative to NaCl alone detected for ChT but not for ChL.

### 2.5. Partial Least Squares Discriminant Analysis (PLS-DA)

**Figure 5:**
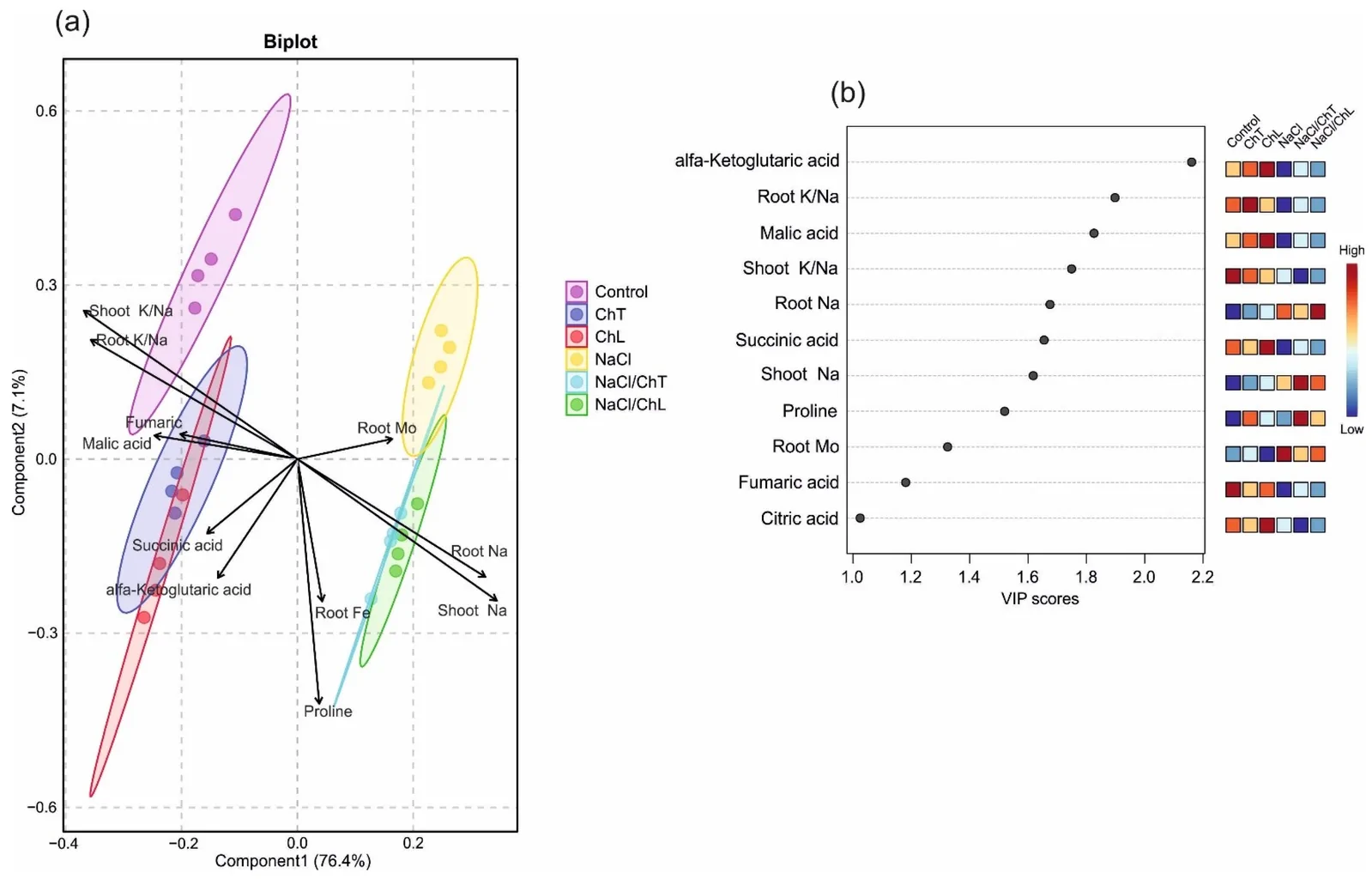
PLS-DA analysis integrating mineral composition, primary metabolites and ionic balance in cucumber exposed to NaCl stress and treated with two chitosan forms (ChT and ChL). PLS-DA biplot showing the distribution of experimental treatments based on the first two latent components (Component 1 = 76.4%, Component 2 = 7.1% of the explained variance). Arrow length reflects the contribution of each variable to the multivariate model, whereas arrow orientation indicates correlations among variables (**a**). VIP scores identify the metabolites and mineral traits with the greatest contribution to treatment separation. Heat maps illustrate the relative abundance of each variable across the experimental treatments (**b**).

PLS-DA analysis integrating mineral composition, primary metabolites, and proline clearly discriminated the experimental treatments, with the first two latent variables explaining 83.5% of the total variance (Component 1, 76.4%; Component 2, 7.1%) (Figure 5a). Variable Importance in Projection (VIP) analysis identified α-ketoglutarate, root K/Na ratio, malate, and shoot K/Na ratio as the variables contributing most strongly to treatment discrimination, whereas citrate showed the lowest discriminatory power among the variables presented (Figure 5b).

The separation of the control and NaCl-treated plants along the first component (76.4% of the explained variance) indicates that excessive salinity constituted the major source of variation among experimental treatments (Figure 5a). NaCl-treated plants clustered with shoot and root Na accumulation, whereas control plants were positively associated with high levels of malate, fumarate, succinate and α-ketoglutarate. Accordingly, plants exposed to NaCl were characterized by reduced concentrations of these organic acids. The K/Na ratio was among the variables with the highest VIP values, indicating its strong contribution to the discrimination of the experimental groups. However, this multivariate importance should not be interpreted as evidence of a significant chitosan-induced restoration of K/Na under saline conditions.

Although Component 2 explained a smaller proportion of the total variance, it consistently differentiated chitosan-treated plants from their respective untreated controls. Irrespective of salinity, plants receiving chitosan (ChL, ChT, ChL + NaCl and ChT + NaCl) were positioned at lower values of Component 2, whereas the control and NaCl samples were located at higher values. This pattern suggests that chitosan induced a reproducible shift in the metabolic and physiological profile that was partially independent of the salt treatment. Notably, the two forms of chitosan occupied somewhat different positions within the saline treatments, suggesting variation in their overall multivariate response patterns. Interestingly, the separation of proline from the cluster of TCA-cycle intermediates may indicate that changes associated with osmotic adjustment and central carbon metabolism are partially independent components of cucumber’s adaptation to salinity (Figure 5a). Moreover, the relatively high VIP score of proline (Figure 5b) suggests that this metabolite contributed substantially to the discrimination among treatments and may represent an important metabolic component of the chitosan-induced response. Although both chitosan forms exerted only minor effects on the concentration of measured mineral elements, they consistently shifted the overall metabolic profile away from the NaCl treatment and towards that of the non-saline controls. Salinity-associated differences were primarily captured by Component 1, whereas Component 2 differentiated chitosan-treated plants from their respective untreated controls. Together, these multivariate results corroborate the physiological and biochemical analyses and support the conclusion that metabolic changes, rather than improved ion exclusion, appear to be a major component underlying the enhanced salinity tolerance in cucumber mediated by chitosan. Hierarchical clustering analysis (Supplementary Figure S1) further confirmed the separation of control and salt-stressed plants and showed that chitosan-treated plants under salinity displayed an intermediate multivariate profile between the NaCl and control groups.

## 3. Discussion

The beneficial effects of chitosan and its derivatives as biostimulants under abiotic stress, including salinity, have been widely reported [10,27,28]. However, such effects seem to be species-specific and depend on many variables, including the salinity level, method of application and physicochemical properties of chitosan [16]. The interpretation of available studies on the application of this biopolymer is also complicated by a lack of methodological standardization. The preparations used in individual experiments differ in molecular weight, degree of deacetylation, raw material source, methods of preparation and dissolution, as well as concentration, exposure time, and method of application. These parameters significantly influence the physicochemical properties and biological activity of chitosan, thereby limiting the direct comparability of results and the formulation of universal conclusions. It is emphasized that the successful implementation of chitosan requires careful consideration of its structural properties and preparation parameters in relation to its specific applications [21,29]. The present study indicates that the biological effects of chitosan under salinity may vary depending on the preparation used, and are associated mainly with metabolic rather than ionic or photosynthetic adjustments.

In several species, chitosan has been shown to alleviate salinity stress partly through the restoration of ion homeostasis. In *Brassica napus*, foliar chitosan application caused reduction in Na accumulation and increased K uptake while enhancing osmolyte synthesis and antioxidant responses [30]. Similarly, chitosan and chitosan nanoparticles increased biomass production and improved mineral homeostasis in salt-stressed *Phaseolus vulgaris* by decreasing Na accumulation and the Na/K ratio while increasing K content, with chitosan nanoparticles showing the strongest effect [31]. Also, foliar applied chitosan increased N, P, K, Mg and Fe, simultaneously reducing Na in *Moringa oleifera* [32]. In contrast, the present study neither ChT nor ChL restored the K/Na ratio or prevented excessive Na accumulation in cucumber roots or shoots. Despite minor trends suggesting a slight improvement in K retention in roots, these effects were not statistically significant. The observed effects were limited to modest, element-specific changes, mainly in P, S, Cu and Zn content, and were generally less pronounced for micronutrients than for macronutrients. This discrepancy may reflect differences in application method (root vs. foliar), species-specific ion transport regulation, or the relatively short exposure time used here. The lack of significant differences among NaCl-containing treatments indicates that, under the present experimental conditions, chitosan did not substantially modify the determined mineral composition—pointing to alternative, non-ionic mechanisms of protection, which are addressed below.

Salinity significantly reduced chlorophyll a, chlorophyll b and carotenoid levels, confirming the sensitivity of the photosynthetic apparatus to NaCl exposure. However, neither chitosan form counteracted this decline, and the differences between NaCl + ChT, NaCl + ChL and NaCl-only treatments were not statistically significant. Chlorophyll fluorescence parameters were comparatively stable. While Fm was significantly influenced by the main effects of salinity and chitosan treatment, no significant salinity × chitosan interaction was detected, and the maximum quantum efficiency of PSII (Fv/Fm) remained unchanged. This suggests that the core photochemical machinery of PSII was not compromised even under salinity, despite the reduction in pigment content. This pattern contrasts with reports in other species, where chitosan clearly protected the photosynthetic apparatus under salt stress. For example, in *Hibiscus syriacus*, chitosan improved photosynthetic efficiency and reduced oxidative damage to the photosynthetic apparatus [33], and comparable protective effects were reported in *Zea mays* [34]. In cucumber, the maintenance of Fv/Fm values despite reduction in pigment contents suggests that photosynthetic protection was not the main manner through which chitosan supported plant growth under salinity in these experimental conditions. Instead, the improved plant tolerance appears to be more closely associated with metabolic and osmotic adjustments.

The concentration of free proline increased more than twofold under salt stress, consistent with its established role as a compatible osmolyte in the plant salt stress response [1]. Also, chitosan application—particularly ChT—further elevated proline accumulation both under salinity and, notably, even in non-stressed plants. The recent study by Bigham Soostani et al. [35] provided molecular evidence that chitosan pre-treatment upregulates genes involved in proline biosynthesis (such as P5CS), priming plants for a stronger defense response under salt stress. This pattern is consistent with a chitosan-induced metabolic response that is at least partly independent of the presence of salinity. A comparable dissociation between ion regulation and osmotic adjustment was reported by Zhang et al. [36] in *Lactuca sativa*, where chitosan increased K content under salinity but did not affect the K/Na ratio while still improving growth, free proline and soluble sugar accumulation, and enhanced antioxidant enzyme activity and reduced membrane lipid peroxidation. These observations are consistent with the concept proposed by Munns and Tester [1], who suggested that the accumulation of compatible osmolytes such as free proline represents an important mechanism of adaptation to osmotic stress and may partially compensate for disturbed ion balance. Similar conclusions were reached by Geng et al. [37], who reported that increased salinity tolerance following chitosan application was associated with changes in sugar and amino acid metabolism.

The present study revealed significant changes in the concentrations of organic acids related to the TCA cycle, which are commonly observed in plants exposed to abiotic factors, including salinity. Together with increased proline accumulation, the obtained results may indicate a reorganization of primary metabolism involving alterations in carbon allocation and redirection of metabolic pathways toward the biosynthesis of stress-related compounds and maintenance of ion and redox homeostasis. These processes are closely interrelated, as the energy and reducing power required for anabolic processes are largely generated through glycolysis and the TCA cycle. These pathways also provide carbon skeletons and intermediates required for the synthesis of numerous classes of specialized metabolites and anabolic pathways [38]. Importantly, the TCA cycle does not function solely as a closed respiratory pathway but constitutes a central metabolic and anaplerotic hub connecting carbon metabolism with nitrogen assimilation and the biosynthesis of amino acids, lipids, and other cellular constituents [39,40]. Consequently, changes in the abundance of individual TCA-cycle intermediates under stress may reflect both altered respiratory metabolism and their differential withdrawal into associated biosynthetic pathways. Organic acids can therefore function as dynamic carbon pools contributing to the maintenance of metabolic and redox homeostasis, although changes in their concentrations alone cannot provide direct information on the direction or magnitude of metabolic fluxes [39].

In the present study, NaCl treatment markedly decreased the concentrations of the measured TCA-related organic acids. Such depletion may result from reduced carbon input into the TCA cycle, increased utilization of particular intermediates in stress-related metabolic pathways, or a combination of these processes [41]. This interpretation may also explain why the response of individual organic acids was not uniform. Citrate, for example, represents an important metabolic branch point linking mitochondrial carbon metabolism with anabolic processes. Following its export from mitochondria, citrate can contribute to the cytosolic acetyl-CoA pool and thereby provide carbon for fatty-acid and other acetyl-CoA-dependent biosynthetic pathways [42,43]. Although increased endogenous citrate accumulation has been reported as part of the response to salinity in some plant species [43,44], the opposite trend was observed in the present study. Moreover, citrate showed a relatively limited response to chitosan in saline conditions. These findings indicate that citrate accumulation is not a universal response to salt stress and may depend on plant species, stress intensity, developmental stage, and the balance between citrate production and its utilization in downstream metabolic pathways.

Particularly noteworthy were the changes in the level of α-ketoglutarate, given its key position at the interface between carbon and nitrogen metabolism. This organic acid provides the carbon skeleton for glutamate biosynthesis and therefore indirectly connects the TCA cycle with the synthesis of several amino acids, including proline. Conversely, proline catabolism generates glutamate, which can subsequently be converted to α-ketoglutarate and re-enter the TCA cycle [45,46]. This metabolic connection is relevant to the present results because salinity strongly increased proline accumulation while decreasing α-ketoglutarate concentration, suggesting substantial redistribution of carbon and nitrogen metabolism under stress. Notably, in the complementary analysis restricted to the saline treatments, ChT increased α-ketoglutarate relative to NaCl alone and its application alone was also associated with substantially enhanced proline accumulation. Rather than demonstrating a direct stimulation of the α-ketoglutarate–glutamate–proline pathway, these simultaneous responses suggest that ChT may influence the coordination between central carbon metabolism and nitrogen-dependent osmotic adjustment under salinity.

In the complementary analysis restricted to the saline treatments, chitosan partially counteracted the salinity-induced depletion of selected TCA-cycle intermediates. Both forms of chitosan caused increase in malate concentrations relative to NaCl, whereas ChT additionally increased α-ketoglutarate and fumarate. These treatment-specific differences were resolved within the uniform saline background, whereas the factorial analysis showed that salinity remained the predominant source of variation for most organic acids. A comparable reorganization of primary metabolism has been reported in *Solanum lycopersicum* treated with a silicon-based biostimulant under excessive salinity [47], where stress acclimation involved changes in the TCA cycle as well as amino acid metabolism. Overall, the present results support an association between chitosan-induced improvement of plant performance under salinity and the modulation of central metabolism; however, metabolic flux analyses would be required to determine whether the observed changes reflect altered synthesis, utilization, or interconversion of individual TCA-cycle intermediates. 

The metabolic changes and responses associated with osmotic adjustment described above were accompanied by changes in plant growth. Salinity reduced shoot and root FW and leaf area, while the two chitosan forms showed different response patterns. In the complementary analysis restricted to saline treatments, ChT significantly increased the analysed growth parameters relative to NaCl alone, whereas the corresponding effects of ChL did not reach statistical significance. Notably, the apparently stronger biological response observed with ChT, despite the better solubility of ChL, suggests that solubility alone may not fully explain the observed responses and that other physicochemical properties, such as molecular weight, charge density, and degree of deacetylation, may also contribute. This is consistent with reports indicating that chitosan-induced growth protection under salinity can occur without full restoration of ion homeostasis, as also shown for *L. sativa* [36] and *Agrostis stolonifera* [37]. Importantly, the observed changes in the accumulation of proline and TCA-cycle intermediates should be interpreted as metabolic responses associated with the improved growth of chitosan-treated plants under salinity rather than as evidence of a specific causal mechanism. Since metabolite concentrations represent the net outcome of their synthesis, utilization, and interconversion, the present data do not allow metabolic fluxes or the direction of carbon redistribution to be determined. Nevertheless, the partial maintenance of selected TCA-related metabolite pools, particularly malate and α-ketoglutarate, together with enhanced proline accumulation, indicates that chitosan treatment was associated with an adjustment of central carbon metabolism under salinity.

Taken together, our results suggest that under the conditions used in this experiment, the observed growth response was more closely associated with osmotic and metabolic adjustments than with changes in ion homeostasis or photosynthetic pigment levels. Importantly, the stability of Fv/Fm suggests that these pigment changes were not accompanied by major impairment of PSII photochemical efficiency. This interpretation is supported by the multivariate PLS-DA and hierarchical clustering analyses. While salinity explained the major source of variation between treatments, chitosan consistently caused an independent shift in the overall physiological and metabolic profile with only minor influences on ion homeostasis. This observation corroborates the physiological and biochemical data and indicates that the growth response to chitosan was more closely associated with changes in primary carbon metabolism and osmotic adjustment than with restoration of ionic balance. This does not exclude a contribution from antioxidant mechanisms, which have been repeatedly implicated in chitosan-mediated salinity resistance in other species, for instance through enhanced polyphenol and flavonoid content and antioxidant enzyme activity in wheat [48] and activation of antioxidant defenses in maize [34]. However, the antioxidant status was not assessed in the present study and remains an open question for cucumber under these conditions. Furthermore, the distinct positioning of ChT- and ChL-treated plants in the multivariate analysis is consistent with differences in their overall response patterns; however, this should not be interpreted as evidence that all individual responses differed significantly between the two forms. Since the preparations differed simultaneously in several physicochemical characteristics and ChL was supplied as the lactate salt, the contribution of individual properties cannot be resolved. Because ChL was applied as the commercially available chitosan oligosaccharide lactate salt, the potential contribution of the lactate counterion cannot be completely separated from that of the chitosan oligomer itself. Thus, the observed differences between ChT and ChL should be interpreted as differences between the two chitosan formulations rather than being attributed exclusively to molecular weight or degree of deacetylation. 

Several limitations should be considered when interpreting the present findings. First, the experiment was conducted using a single cucumber cultivar, one salinity level and one concentration of each chitosan preparation. Although the selected NaCl concentration was based on preliminary dose–response experiments, a broader range of salinity levels and chitosan concentrations would be required to establish dose-dependent responses. Second, the two chitosan preparations were characterized primarily according to manufacturer-provided molecular weight and degree of deacetylation; therefore, the contribution of individual physicochemical properties to their differential biological activity cannot be resolved. Third, all measurements were performed after 14 days of treatment, and consequently the temporal dynamics of the observed responses remain unknown. Fourth, antioxidant status, oxidative damage and molecular signalling were not directly assessed, limiting mechanistic interpretation. Finally, some mineral analyses were based on a relatively small number of biological replicates, which may contribute to substantial biological variability for individual elements. Further studies incorporating time-course experiments, antioxidant and oxidative-stress measurements, appropriate formulation controls, and integrated metabolomic and transcriptomic approaches are required to elucidate the mechanisms underlying these responses.

## 4. Materials and Methods

### 4.1. Plant Materials, Growth Conditions, and Experimental Design

Seeds of cucumber (*Cucumis sativus* L.) cv. Polan F1 germinated in moist quartz sand for 8 days at 25 °C in an environment-controlled phytotron. Uniform seedlings at the two-true-leaf stage were transferred to 1 L containers filled with Hoagland’s II nutrient solution (pH 5.5). Plants were cultivated in a growth chamber (Sanyo MLR-350HT, Sanyo Electric Co., Ltd., Oizumi, Japan) under the following controlled conditions: photosynthetic photon flux density (PPFD) of 250–270 µmol m^−2^ s^−1^, a 14 h light/10 h dark photoperiod, day/night temperature of 25/22 °C, and relative humidity of 60–65%. After a 3-day acclimation period in the liquid nutrient medium, salinity and chitosan treatments were imposed.

The NaCl concentration used in the main experiment was selected based on preliminary dose–response trials conducted on cucumber cv. Polan F1 under identical laboratory conditions. In these pilot experiments, a range of NaCl concentrations was evaluated to identify a level that induced clear but non-lethal stress symptoms, thereby enabling the assessment of stress-alleviating treatments. The tested NaCl concentrations were 0, 30, 40, 50, 60 and 70 mmol L^−1^, corresponding to electrical conductivity (EC) values of 2131, 5475, 6494, 7344, 8584 and 9734 µS cm^−1^, respectively. A concentration of 50 mmol L^−1^ NaCl caused reproducible reductions in growth and physiological performance without inducing severe tissue damage or plant mortality. Therefore, this level was selected as a non-lethal salinity stress for further experiments.

The concentration of chitosan (10 mg L^−1^) was selected based on previous reports demonstrating the biological effectiveness of low-dose chitosan applications in hydroponic systems. Several studies have shown that chitosan in the range of 5–50 mg L^−1^ can effectively modulate plant stress responses without negatively affecting growth [29,32,34]. Therefore, a moderate concentration of 10 mg L^−1^ was chosen as a physiologically relevant dose that allows evaluation of stress-mitigating effects while minimizing the risk of non-specific growth inhibition.

The experimental design included two salinity levels (0 mmol L^−1^ NaCl as control and 50 mmol L^−1^ NaCl) and two chemically distinct forms of chitosan: high-molecular-weight chitosan (ChT) and chitosan oligosaccharide lactate (ChL), both applied at a concentration of 10 mg L^−1^ of the nutrient solution. The chitosan stock solution (500 mg L^−1^, pH = 4.0) was prepared in advance, and 20 mL of the stock solution was added per liter of nutrient medium in the respective treatments. Consequently, there were six treatments, with four containers per treatment, three plants per container, resulting in 12 plants per treatment.

Standard high-molecular-weight chitosan from shrimp shells (ChT; C3646, Sigma-Aldrich, St. Louis, MO, USA), with a MW of 190,000–375,000 g mol^−1^ and ≥75% degree of deacetylation, and chitosan oligosaccharide lactate (ChL; 523682, Sigma-Aldrich, St. Louis, MO, USA), with an average MW of approximately 5000 g mol^−1^ and >90% degree of deacetylation, were used. The molecular weight values of chitosan were obtained from the manufacturer’s technical information. Plants were grown under differentiated conditions for 14 days. At the end of the experimental period, the growth parameters and physiological and biochemical analyses were performed.

### 4.2. Determination of Plant Growth and Physiological Parameters

Nine plants were harvested from each treatment and separated into roots and shoots. The fresh weight (FW) of the organs was determined individually. The leaf area of the second true leaf from the bottom of each plant was measured using a laser area meter (CI-202, CID Bio-Science, Camas, WA, USA).

Chlorophyll a fluorescence was assessed using a Handy-PEA portable photofluorimeter (Hansatech Instruments, King’s Lynn, UK) by determining the maximum fluorescence (Fm), minimum fluorescence (Fo), and the maximum quantum efficiency of photosystem II expressed as the Fv/Fm ratio. The Fv/Fm parameter is widely used as a reliable indicator of the maximum photochemical efficiency of PSII in dark-adapted leaves [49]. Prior to measurements, selected leaf fragments were dark-adapted for 15 minutes using dedicated clips, after which fluorescence parameters were recorded.

Samples for photosynthetic pigment determination were collected from the second true leaf from the bottom and homogenized in 80% (*v*/*v*) acetone using a mortar and pestle. The homogenate was filtered under vacuum and rinsed with additional acetone to ensure quantitative pigment recovery. The combined extracts were transferred to volumetric flasks and adjusted to a defined volume with acetone. Absorbance was measured at 663, 645, and 470 nm (Cecil CE 9500, Cecil Instruments, Cambridge, UK), and pigment concentrations were calculated according to the equations provided by Lichtenthaler and Wellburn [50].

To estimate the levels of free proline, the method of Bates et al. [51] was employed. Leaf samples (0.5 g) were ground in 3% sulfosalicylic acid and filtered. Aliquots of the filtrate were reacted with equal volumes of glacial acetic acid and acid ninhydrin for 1 h at 100 °C. Following incubation, the tubes were rapidly cooled. The chromophore was extracted using toluene, and its absorbance was measured spectrophotometrically at 520 nm (Cecil CE 9500) with pure toluene as a blank. Final values were calculated using a proline standard and reported in µg g^−1^ FW.

### 4.3. Identification and Quantitative Analysis of Organic Acids

Samples for determining acids related to the TCA cycle were taken from the second true leaf from the bottom. The quantification of organic acids in cucumber leaf extracts was performed using high-performance liquid chromatography (HPLC) equipped with a diode array detector (DAD) (VWR Hitachi Chromaster 600, Merck, Darmstadt, Germany). Chromatographic separation was achieved on a Rezex ROA-Organic Acid H^+^ (8%) column (300 × 7.8 mm; Phenomenex, Torrance, CA, USA). The mobile phase consisted of 0.0025 M H_2_O_4_, delivered isocratically at a flow rate of 0.6 mL min^−1^. The column temperature was maintained at 40 °C, and the injection volume was 10 µL.

Detection was carried out at λ = 210 nm, and quantification of malic acid, citric acid, fumaric acid, α-ketoglutaric acid, and succinic acid was based on external calibration curves prepared from analytical standards (Sigma-Aldrich, Merck KGaA, Darmstadt, Germany). The identification of organic acids in the samples was confirmed by comparing retention times and UV spectra with those of authentic standards. The results were expressed as mg g^−1^ FW.

### 4.4. Determinations of Mineral Elements

Samples were digested in closed DigiTUBEs (SCP Science, Baie-D’Urfe, QC, Canada) in a heating block (DigiPREP, SCP Science, Baie-D’Urfe, QC, Canada) in the following two steps. Root samples were mixed with 5 mL of 65% HNO_3_ Suprapur (Sigma-Aldrich), and shoots were mixed with 2 mL of deionized water, 8.5 mL of 65% HNO_3_ Suprapur and 1 mL of 30% H_2_O_2_. The first step of digestion was performed at 110 °C for 120 minutes. After cooling the solutions to room temperature, 2 mL of 65% HNO_3_ was added to each sample, and the second step was performed at 110 °C for 60 minutes. Samples were filtered through DigiFILTERs (SCP Science, Baie-D’Urfe, QC, Canada), spiked with yttrium internal standard and filled up to 50 mL with deionized water in DigiTUBEs (SCP Science, Baie-D’Urfe, QC, Canada).

Analysis of sodium, potassium, magnesium and calcium was performed using high-resolution inductively coupled plasma optical emission spectroscopy (HR-ICP-OES) PlasmaQuant PQ 9000 Elite (Analytik Jena AG, Jena, Germany). The following wavelengths were used for elements quantification: Na—589.5924 nm, K—766.4911 nm, Mg—285.2126 nm, Ca—315.8869 nm and Y—371.0300 nm. The signal reading time was 3 s; the plasma monitoring radial direction was for Na and K and attenuated axially for Mg and Ca. RF generator power and plasma, auxiliary and nebulizer gases flows were as follows: 1300 W and 12.0, 0.50, 0.60 L/min. The upper measurement ranges for Na, K, Mg and Ca were respectively 200, 400, 20 and 100 mg/L. Validity of calibration was verified using an ISO 17294 Tuning Solution A (SCP Science, Baie-D’Urfe, QC, Canada). Analysis of Ca and Mg in all samples and Na and K in selected shoot samples was carried out in solutions diluted 25 times due to concentrations of analytes above the upper measurement ranges. Each measurement was performed in triplicate. Technical replicate measurements were averaged before statistical analysis and were not treated as independent biological replicates. The trueness of the elements determination results was checked by analyzing the certified matrix reference materials: Bovine Liver 1577c (NIST, Gaithersburg, MD, USA) and Soya Bean Flour INCT-SBF-4 (INCT, Warsaw, Poland).

### 4.5. Statistical Analysis

The study was conducted using a completely randomized experimental design. All data were first subjected to two-way ANOVA to evaluate the effects of salinity (0 and 50 mM NaCl), chitosan treatment (control, ChT and ChL) and their interaction. Subsequently, for selected parameters where salinity constituted the predominant source of variation, a complementary one-way ANOVA was performed exclusively on the saline treatments (NaCl, NaCl + ChT and NaCl + ChL). This analysis was intended to resolve treatment-specific responses that could be obscured by the strong main effect of salinity. The results of the one-way ANOVA are presented only for variables for which this complementary analysis revealed additional statistically significant differences among the NaCl treatments. For all remaining variables, where the conclusions drawn from one-way and two-way ANOVA were consistent, no additional one-way ANOVA results are presented. When significant effects were detected, mean values were compared using Tukey’s post hoc test at *p* < 0.05. Statistica ver. 13.3 software (TIBCO Software Inc. 2017, Palo Alto, CA, USA) was used for all statistical analysis.

Partial least squares-discriminant analysis (PLS-DA) was performed on MetaboAnalyst 6.0 platform (www.metaboanalyst.ca, accessed on 3 August 2026) using mean-centered and unit variance-scaled data. The data matrix was constructed at the experimental unit level using three independent containers per treatment, matched across the variables included in the analysis. When multiple measurements were available within a container, they were averaged to obtain a single container-level value. Thus, each row of the data matrix represented one independent experimental unit and contained the corresponding values for all variables included in the multivariate analysis. To facilitate biological interpretation, the 11 variables with the highest variable importance in projection (VIP) scores were selected for visualization. Sample discrimination was assessed using the score and loading plots, while the contribution of individual variables was evaluated based on their VIP scores. 

## 5. Conclusions

Our results demonstrate that the two chitosan preparations evoked partially different response patterns in cucumber exposed to salinity. ChT, but not ChL, significantly improved shoot and root fresh weight and leaf area under salt stress, despite the persistence of salinity-induced reductions in photosynthetic pigment content. The stability of Fv/Fm indicates that these pigment changes were not accompanied by a significant alteration in the maximum quantum efficiency of PSII. The contrasting responses of photosynthetic pigments, chlorophyll fluorescence, mineral composition and primary metabolites were more consistent with effects on metabolic rather than photochemical processes, highlighting the complexity of chitosan action under salinity stress. Salt stress substantially depleted the pool of major TCA-cycle intermediates, particularly malate and α-ketoglutarate, indicating disruption of central carbon metabolism. Interestingly, both chitosan formulations partially alleviated the NaCl-induced decline in malate, whereas ChT additionally increased α-ketoglutarate and fumarate under saline conditions, despite having only limited effects on mineral composition and no substantial effect on the K/Na ratio. These findings suggest that the improved growth observed following ChT application under salinity was associated with changes in selected metabolic traits rather than with restoration of mineral status. This interpretation is further supported by the consistency between univariate and multivariate analyses. However, the present data do not establish a causal mechanism. Overall, the results indicate that the response to chitosan varied according to the preparation applied and highlight the importance of considering the physicochemical characteristics and chemical formulation of chitosan preparations when evaluating their potential as plant biostimulants.

## Figures and Tables

**Table 1:** Effect of chitosan application on mineral composition of roots of *C. sativus* grown under NaCl-induced stress.

Treatment	Macronutrient Contents (mg g^−1^ DW)					Micronutrient Contents (µg g^−1^ DW)					Na (mg g^−1^ DW)	K/Na Ratio
	K	Ca	P	Mg	S	Fe	Zn	Cu	Mn	Mo		
Control	104.04 ±2.03 a	7.55 ±1.28 a	7.80 ±0.29 b	2.20 ±0.21 a	6.68 ±0.52 b	1531 ±179 b	571.4 ±98.6 b	28.59 ±0.59 b	63.53 ±2.38 b	0.70 ±0.03 a	0.75 ±0.03 c	138.7 ±2.9 a
ChT	111.23 ±5.20 a	9.20 ±1.80 a	10.13 ±1.17 a	2.00 ±0.28 a	7.99 ±1.37 b	2711 ±1136 ab	742.1 ±158 ab	36.62 ±11.16 b	73.97 ±19.10 b	0.88 ±0.01 a	0.79 ±0.03 c	141.2 ±5.0 a
ChL	107.98 ±2.70 a	8.79 ±1.13 a	8.77 ±0.31 b	1.91 ±0.16 a	6.68 ±0.60 b	2754 ±1053 ab	662.7 ±54 b	32.77 ±1.36 b	79.83 ±4.10 b	0.72 ±0.04 a	0.89 ±0.11 c	122.7 ±15.3 a
NaCl	46.97 ±1.49 b	7.45 ±0.52 a	10.91 ±0.35 a	1.62 ±0.07 b	10.89 ±0.26 a	2436 ±430 ab	850.8 ±162 a	76.69 ±6.75 a	116.91 ±3.13 a	0.46 ±0.08 b	42.95 ±0.67 a	1.09 ±0.02 b
NaCl + ChT	51.28 ±3.35 b	6.94 ±0.09 a	11.05 ±0.35 a	1.61 ±0.05 b	10.14 ±0.55 a	3014 ±283 ab	954.1 ±42 a	71.90 ±5.60 a	117.56 ±5.85 a	0.77 ±0.07 a	42.63 ±2.11 a	1.18 ±0.07 b
NaCl + ChL	49.70 ±2.77 b	7.69 ±0.63 a	10.66 ±1.00 a	1.76 ±0.08 ab	9.83 ±0.29 a	3188 ±563 a	1039.7 ±178 a	82.12 ±7.16 a	114.75 ±12.29 a	0.68 ±0.07 a	43.81 ±1.68 a	1.13 ±0.04 b
Two-way ANOVA												
Salinity	***	*	**	***	***	*	ns	***	*	*	***	***
Chitosan	ns	ns	ns	ns	*	*	ns	ns	ns	ns	ns	ns
Salinity × Chitosan	ns	ns	ns	ns	ns	ns	ns	ns	ns	*	ns	ns

Two-way ANOVA: ns, not significant; * *p* < 0.05; ** *p* < 0.01; *** *p* < 0.001. Means (± SD, *n* = 3) within a column sharing the same letter do not differ significantly according to Tukey’s test at *p* < 0.05.

**Table 2:** Effect of chitosan application on mineral composition of shoots of C. *sativus* grown under NaCl-induced stress.

Treatment	Macronutrient Contents (mg g^−1^ DW)					Micronutrient Contents (µg g^−1^ DW)					Na (mg g^−1^ DW)	K/Na Ratio
	K	Ca	P	Mg	S	Fe	Zn	Cu	Mn	Mo		
Control	52.24 ±2.14 a	43.14 ±0.78 a	11.89 ±0.35 a	9.82 ±0.41 a	10.35 ±0.39 a	99.45 ±0.8 ab	238.65 ±5.5 a	11.86 ±1.42 a	44.93 ±0.28 a	0.28 ±0.01 a	0.31 ±0.03 b	170.5 ±11.6 a
ChT	52.65 ±2.58 a	44.37 ±2.77 a	12.19 ±0.25 a	10.46 ±0.88 a	10.54 ±0.29 a	107.78 ±14.3 a	242.66 ±24.1 a	11.10 ±1.02 a	50.45 ±3.19 a	0.20 ±0.03 a	0.31 ±0.03 b	173.2 ±16.75 a
ChL	51.46 ±0.32 a	43.24 ±0.96 a	11.95 ±0.17 a	9.82 ±0.65 a	10.63 ±0.07 a	104.52 ±21.6 a	229.06 ±6.1 a	10.97 ±0.63 a	44.90 ±1.26 a	0.18 ±0.00 a	0.31 ±0.00 b	168.8 ±1.3 a
NaCl	27.54 ±1.22 b	20.99 ±0.72 c	10.40 ±0.29 b	7.76 ±0.13 b	10.06 ±0.24 a	100.11 ±14.7 ab	225.39 ±11.4 a	11.39 ±0.42 a	42.92 ±5.19 a	0.12 ±0.02 b	38.86 ±2.17 a	0.71 ±0.02 b
NaCl + ChT	27.69 ±1.74 b	25.60 ±0.85 b	10.95 ±0.21 ab	7.72 ±0.10 b	10.10 ±0.55 a	79.61 ±3.3 b	242.50 ±19.5 a	13.58 ±0.38 a	50.09 ±2.49 a	0.08 ±0.01 b	38.66 ±3.70 a	0.70 ±0.02 b
NaCl + ChL	26.76 ±1.79 b	26.80 ±2.11 b	10.56 ±0.26 b	8.15 ±0.13 b	9.99 ±0.17 a	109.68 ±4.6 a	233.66 ±12.7 a	12.11 ±1.38 a	46.82 ±1.94 a	0.09 ±0.01 b	39.97 ±0.65 a	0.67 ±0.05 b
Two-way ANOVA												
Salinity	***	***	**	***	ns	ns	ns	*	ns	***	***	***
Chitosan	ns	ns	ns	ns	*	ns	ns	ns	ns	ns	ns	ns
Salinity × Chitosan	ns	*	ns	ns	ns	ns	ns	ns	ns	ns	ns	ns

Two-way ANOVA: ns, not significant; * *p* < 0.05; ** *p* < 0.01; *** *p* < 0.001. Means (± SD, *n* = 3) within a column sharing the same letter do not differ significantly according to Tukey’s test at *p* < 0.05.

## Data Availability

The raw data supporting the conclusions of this article will be made available by the authors on request.

## Supplementary Materials

The following supporting information can be downloaded at https://www.mdpi.com/article/10.3390/molecules31183306/s1. Figure S1: Hierarchical clustering heatmap of physiological, biochemical, and mineral traits in *C*. *sativus* grown under control and NaCl-stress conditions with or without chitosan (ChT or ChL). The heatmap represents the relative distribution of measured traits in individual biological replicates, with samples and variables hierarchically clustered based on similarity. The color scale shows the relative level of each variable, with blue representing lower values and red representing higher values. The upper annotation shows the experimental treatment: Control, ChT, ChL, NaCl, NaCl + ChT, and NaCl + ChL.
