# Supplementary material for: Comparative Effects of High-Molecular-Weight Chitosan and Chitosan Oligosaccharide Lactate on Metabolic Adjustment and Salinity Tolerance in Cucumis sativus L
Source: Molecules. 2026 Sep 17;31(18):3306. doi: 10.3390/molecules31183306 (PMC13610316; doi:10.3390/molecules31183306)
Supplement: Supplementary file 1 [file molecules-31-03306-s001.zip › molecules-4552149-supplementary.pdf]

SUPPLEMENTARY MATERIALS

Comparative Effects of High-Molecular-Weight Chitosan and Chitosan Oligosaccharide Lactate on Metabolic Adjustment and Salinity Tolerance in *Cucumis sativus* L.

Barbara Hawrylak-Nowak [1], Maria Stasińska-Jakubas [1,*], Jan Sawicki [2], Renata Matraszek-Gawron[1], Weronika Jarmulska [1], Magdalena Czelej-Wrzesień [3], Sławomir Dresler [2,4]

[1] Department of Botany and Plant Physiology, University of Life Sciences in Lublin, Akademicka 15, 20-950 Lublin, Poland; barbara.nowak@up.edu.pl (B. H-N.); renata.matraszek@up.edu.pl (R.M-G.); weronika.jarmulska@up.edu.pl (W.J.)
[2] Department of Analytical Chemistry, Medical University of Lublin, Chodźki 4a, 20-093 Lublin, Poland; jan.sawicki@umlub.edu.pl (J.S); slawomir.dresler@umlub.edu.pl (S.D.)
[3] Department of Vascular Surgery and Angiology, Medical University of Lublin, Staszica 16, 20-081 Lublin, Poland; magdalena.czelej@umlub.edu.pl (M.C-W.)
[4] Department of Plant Physiology and Biophysics, Maria Curie-Skłodowska University, Akademicka 19, 20-033 Lublin, Poland

* Correspondence: maria.jakubas@up.edu.pl

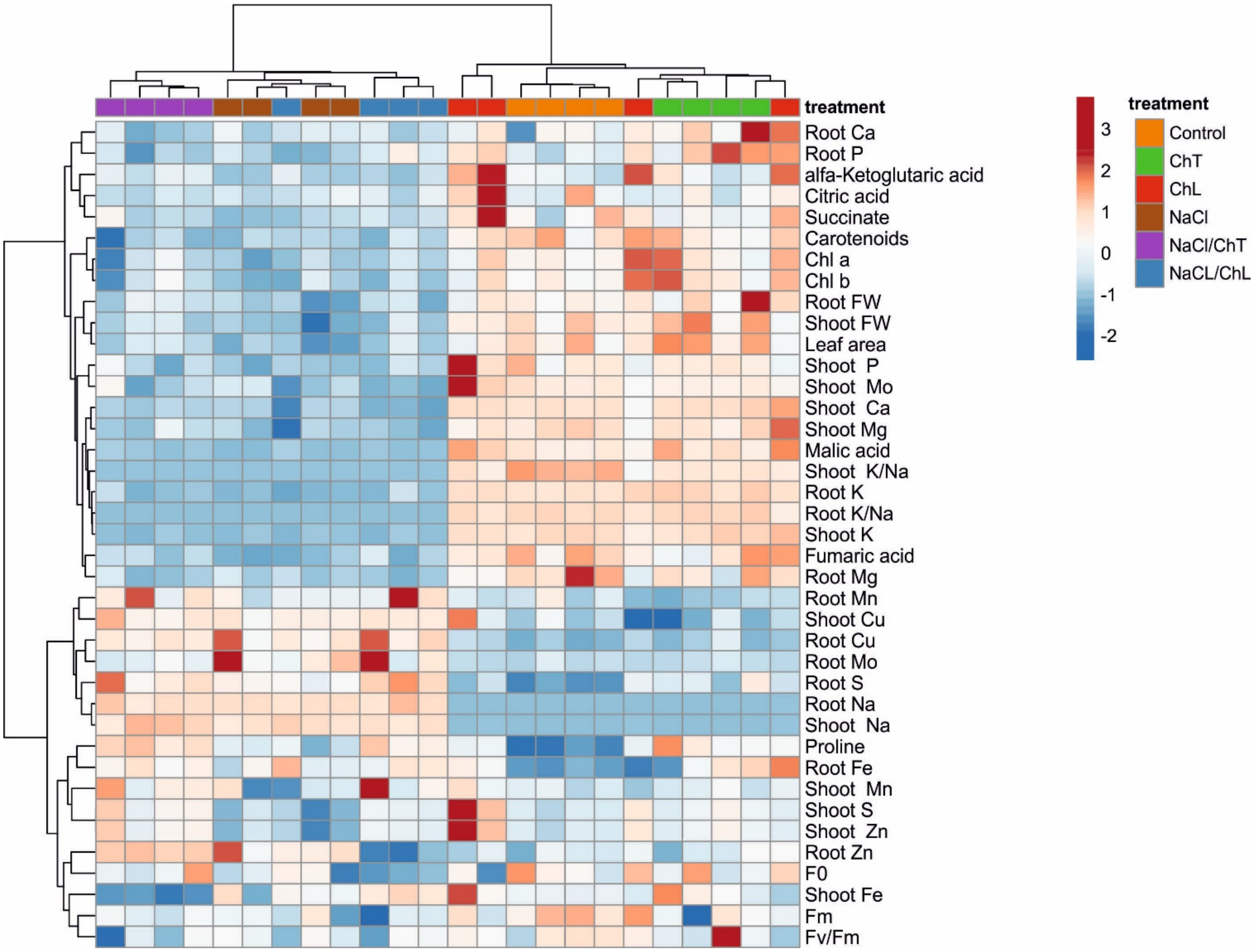

Figure S1. Hierarchical clustering heatmap of physiological, biochemical, and mineral traits in *C. sativus* grown under control and NaCl-stress conditions with or without chitosan (ChT or ChL). The heatmap represents the relative distribution of measured traits in individual biological replicates, with samples and variables hierarchically clustered based on similarity. The color scale shows the relative level of each variable, with blue representing lower values and red representing higher values. The upper annotation shows the experimental treatment: Control, ChT, ChL, NaCl, NaCl+ChT, and NaCl+ChL.
